# Comparison of Bromelain and Diclofenac in the Management of Postoperative Pain and Quality of Life Following Root Canal Therapy: A Randomized Controlled Trial

**DOI:** 10.7759/cureus.71995

**Published:** 2024-10-21

**Authors:** Akbarbasha Sherin, Subha Anirudhan, Minu Koshy

**Affiliations:** 1 Conservative Dentistry and Endodontics, Sri Ramakrishna Dental College and Hospital, Tamil Nadu Dr. Maruthur Gopalan Ramachandran (MGR) University, Coimbatore, IND

**Keywords:** bromelain, nsaids, postoperative pain, quality of life, root canal therapy, visual analog scale

## Abstract

Aim

This study aimed to compare the analgesic efficacy and adverse effects of oral bromelain and diclofenac in relieving postoperative pain and improving postoperative quality of life in patients with irreversible pulpitis in mandibular first molars after root canal therapy.

Materials and methods

A simple randomized double-blinded clinical trial was carried out. One hundred patients with symptomatic pulpitis requiring root canal therapy of mandibular first permanent molars were randomly divided into two groups after obtaining informed consent. After access opening, preparation of the root canal and temporization, Group I (n=50) patients received oral bromelain 200 mg and Group II (n=50) received oral diclofenac sodium 50 mg, respectively. The patients were asked to rate their pain on a visual analog pain intensity scale (VAS) and rate their quality of life using the postoperative quality of life (POQoL) questionnaire after 6, 12, 24, 48, and 72 hours.

Results

Bromelain and diclofenac were equally effective for postoperative analgesia, but diclofenac showed immediate pain relief and improved quality of life at 6 hours. After 12 hours bromelain showed similar effects in managing pain and postoperative quality of life with less adverse effects.

Conclusions

When compared to diclofenac, bromelain has the same analgesic efficacy after 12 hours but a lower risk of adverse effects in patients receiving root canal therapy for postoperative pain. Diclofenac is more effective in immediate postoperative pain relief and improvement in quality of life for patients with moderate to severe pain.

## Introduction

Interappointment pain is an often-encountered adverse effect of endodontic treatment. Root canal therapy damages the pulp and peri-radicular tissues mechanically, chemically, and microbiologically. Over-instrumentation, periapical irrigant or medicament extrusion, and restoration with high points are all critical factors that frequently induce inflammation, resulting in an acute episode in the middle of treatment [1]. The pain may begin within a couple of hours and linger for as long as 48 hours [2]. Anxiety, apprehension about dental treatment, and other psychological variables can all alter pain perception and reaction levels in patients [3]. More than 80% of people who suffered from preoperative pain also had considerable interappointment and postoperative pain after root canal therapy [3,4].

Nonsteroidal anti-inflammatory medicines (NSAIDs) such as diclofenac sodium are frequently recommended to alleviate postoperative problems, notably pain. The use of NSAIDs, on the other hand, is usually associated with a spectrum of side effects, most of which are connected to the dermatologic, hematologic, renal, and gastrointestinal systems [5].

Bromelain is a natural, fluid extract of pineapple stems and its immature fruits that contain a complex combination of thiol-endopeptidases and is extracted by the process of centrifugation, ultrafiltration, and lyophilization [6,7]. In contrast to NSAIDs, bromelain causes selective inhibition of proinflammatory thromboxane synthesis and alters the thromboxane/prostacyclin (PGI2) ratio in favor of anti-inflammatory prostacyclin [8]. Due to this mechanism, oral bromelain has exhibited effects such as analgesia, anti-edema, anti-inflammatory, and fibrinolysis, without causing the common adverse effects of NSAIDs [9]. Bromelain in any form exhibits complete absorption and there are no intestinal degradation problems [7]. It has recently been utilized as an anti-inflammatory medicine in orthopedics, plastic surgery, oral surgery, and endodontics [10].

Literature is scarce regarding the use of bromelain for post-endodontic pain management in comparison to the commonly used NSAIDs. Hence, our research aimed to compare the analgesic efficacy in terms of postoperative pain, improvement in quality of life, and adverse reactions of bromelain and diclofenac given orally after root canal therapy.

## Materials and methods

Study design

**Figure 1:**
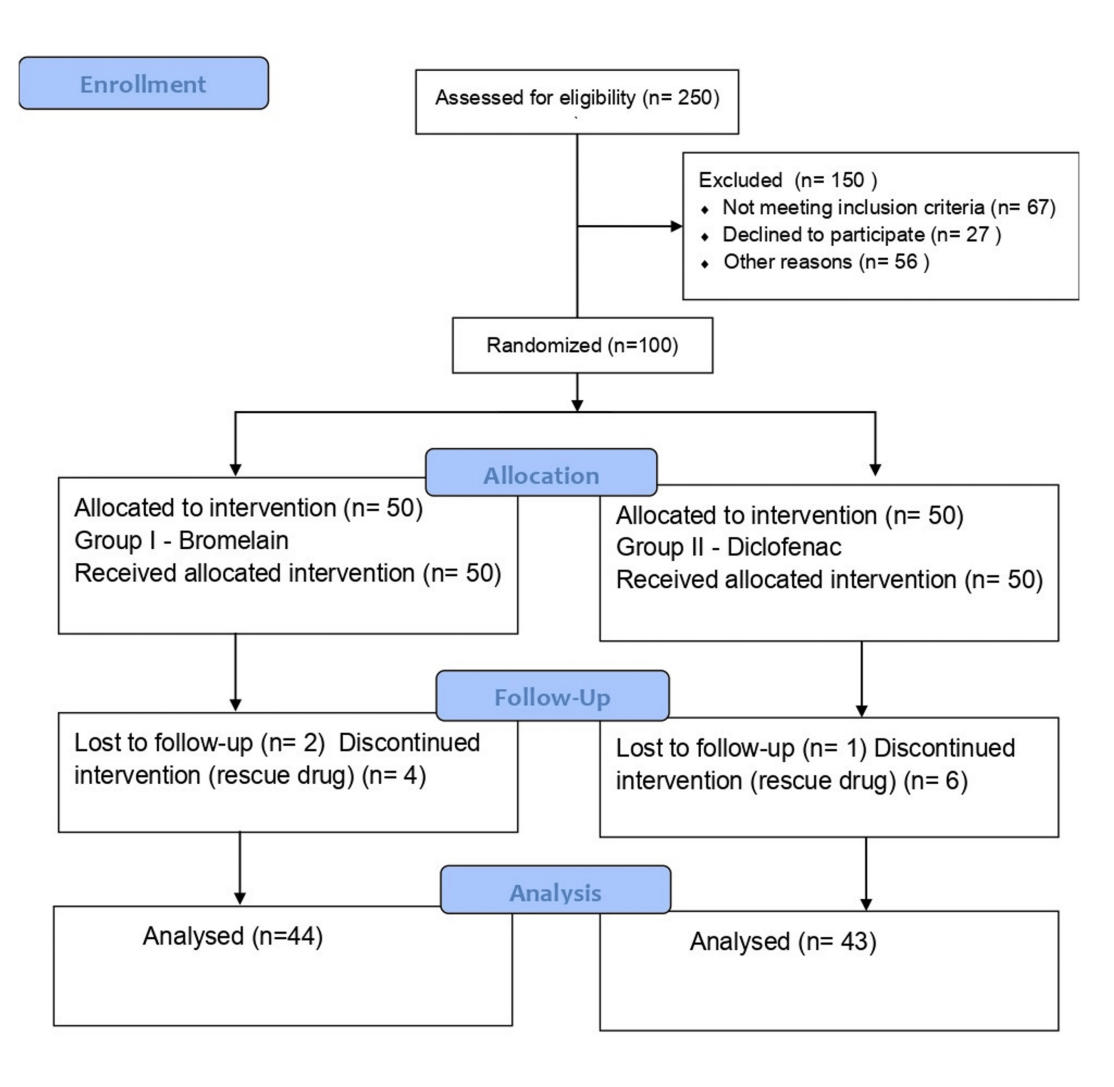
CONSORT flow diagram The diagram was created by the authors of this article.

This single-centered, parallel-group, double-blinded randomized clinical trial was carried out conforming to the criteria of the institutional ethics committee and followed CONSORT guidelines from 1/08/2023 to 1/02/2024 (Figure 1). The study was designed and executed in the Department of Conservative Dentistry and Endodontics, Sri Ramakrishna Dental College and Hospital, Coimbatore, TN, India. Prior to commencement, the study was registered in the Clinical Trials Registry of India (CTRI Reg. No. CTRI/2023/055392). Before commencing the treatment of root canals, each patient submitted informed and signed consent in accordance with ethical norms. The primary outcomes were the assessment and comparison of postoperative pain at 6,12, 24,48, and 72 hours using the visual analog scale (VAS) and quality of life at the same time intervals using the postoperative quality of life (POQoL) questionnaire. The secondary outcomes assessed were any adverse effects reported by the patients during the trial period and the need to take rescue drugs for pain relief.

Sample size

The minimal sample size required for determining a difference of 30% that was clinically significant in postoperative quality of life and post-endodontic pain between the experimental groups was 80 individuals, with a type I error of 0.05 and a statistical power of 80% [11]. To account for exclusions and dropouts, the number of candidates involved in this research was increased to 100. The PS software, version 3.1.2 (https://biostat.app.vumc.org/wiki/Main/PowerSampleSize ), was used to evaluate the power and sample size.

Inclusion and exclusion criteria

This investigation pertains to initial root canal therapy performed on permanent mandibular first molars in adults aged between 18 and 60 years exhibiting symptomatic irreversible pulpitis (complaint of pain in the affected tooth, presence of deep caries, exaggerated response to cold and electric pulp tests). Inclusion criteria were cases demonstrating symptomatic presentation of moderate to severe pain in permanent mandibular first molars, robust general health, and possessing an adequate crown structure conducive to isolation. Conversely, exclusion criteria comprised instances featuring radiographic evidence of periapical radiolucency or sinus tract, pregnancy, history of smoking, teeth that were not amenable to treatment or restoration, pronounced periodontal disease, canals exhibiting intricate morphology or calcification, presence of internal or external resorption, abscess or cellulitis of dental origin, patients manifesting parafunctional habits, individuals contending with chronic systemic ailments, and those who recently took analgesics or antibiotics within a 6-hour timeframe preceding therapy initiation. This delineation of criteria ensured a meticulously selected sample, thereby augmenting the study’s precision and validity.

Out of 250 outpatients, 100 patients reporting pain in the mandibular molar region and with diagnosis of symptomatic irreversible pulpitis, with or without apical periodontitis, requiring endodontic therapy were selected according to inclusion criteria and divided into two groups by randomization viz., Group I (n=50) - bromelain 200 mg (Healthy Hey Foods LLP) and Group II (n=50) - diclofenac 50 mg, respectively. The randomization sequence was generated in randomization.org using block randomization by a research assistant and the allocation written on a card was concealed in opaque envelopes. To reduce or decrease individual variability in treatment across clinicians, all diagnostic and treatment procedures were performed by a lone operator.

Procedure

Each patient was explained about and asked to rate his or her pain on a VAS of pain intensity on a scale of 1 - 10 before anesthesia of the treatment area [12]. Only those patients with a VAS pain score of 4 or above (i.e. moderate to severe pain) were recruited. The access of the concerned tooth was opened under inferior alveolar nerve block local anesthesia (1.8 ml of 2% lignocaine with 1:80,000 epinephrine), and the canals were cleaned and shaped after working length determination (electronic apex locator and periapical radiographs) with ProTaper rotary endodontic files (Dentsply Maillefer) up to F2 size for mesial canals, F2 in case of two distal canals and F3 for single distal canals. Canal irrigation was done with saline and 5.25% Sodium Hypochlorite (Coltene CanalPro) using a 27-gauge side vented needle. The volume of hypochlorite used was 2 ml in between instrumentation and 4 ml/canal for 1 minute for final irrigation. After the final rinse with normal saline, the canals were completely dried with paper points, and a sterile cotton dressing was placed before interim restoration (3M ESPE Cavit-G temporary filling material). After completion of the procedure, a card indicating the postoperative drug was retrieved from the envelope. Group I received 200 mg bromelain (HealthyHey Nutrition) orally and Group II received 50 mg diclofenac (Voveran, Novartis India Ltd.) orally immediately following root canal therapy [11]. The drugs were prescribed to be taken twice a day for three days. Both the patients and operator were blinded to the drug they were dispensed.

Patients were explained about VAS scores and POQoL and a printed response form with these scales was handed to the patients with instructions to indicate the same at different time intervals. In addition, patients were explained about and asked to record any adverse effects. Ibuprofen 400 mg was given as a rescue drug, to be prescribed by the investigator when contacted by the patient in case of unresolved pain. The patients were called on the phone to enquire and remind them to write their pain scores also. One impartial investigator blinded to the groups evaluated the degree and intensity of pain with the aid of a VAS after 6 hours, 12 hours, 24 hours, 48 hours, and 72 hours at the next appointment. POQoL questionnaires were used to assess the quality of life after 6 hours, 12 hours, 24 hours, 48 hours, and 72 hours; the patients chewing, speaking, sleeping, everyday functioning, and maintaining social relations were assessed. Data collected were compiled and statistically evaluated.

Statistical analysis

SPSS Statistics version 21 (IBM Corporation, SPS Inc., Chicago, IL, USA) was used for statistical analysis. For continuous data, the results were reported as Mean (SD), and for categorical measurements, as frequency (%). The Shapiro-Wilk tests were utilized to determine the normality of variables. Since the data did not follow a normal distribution, the Mann-Whitney U test and the chi-square test were used to ascertain the difference between the two groups. A significant p-value was defined as less than 0.05.

## Results

One hundred patients were enrolled in this study and randomly distributed to the study groups. Of the 50 patients in Group I, data were used from only 44 patients as two patients did not report back and four patients took the rescue medication. In Group II, one patient did not report back while six patients took the rescue medication and hence were excluded from the study. Thus, we used data from the remaining 43 patients from this group for statistical evaluation. The baseline characteristics of patients recruited for the study are given in Table 1. The average preoperative pain score of the patients in Group I was 6.4, whereas in Group II was 6.1 and there was no significant difference between these baseline values. There was a marked depletion in postoperative pain in patients in both groups compared to preoperative pain.

**Table 1:** Baseline characteristics of the study participants in Group I and Group II

Demographic Data	Group 1	Group 2
Sex		
Male	32	40
Female	18	10
Mean age	42.3 +/- 4.08	38.7+/- 3.22
Age-wise Distribution		
18-30 years	12	08
30-40 years	21	16
40-50 years	13	16
50-60 years	04	10
Tooth		
Left mandibular first molar	21	30
Right mandibular first molar	29	20
Mean preoperative pain score	6.4	6.1

Primary outcome

**Figure 2:**
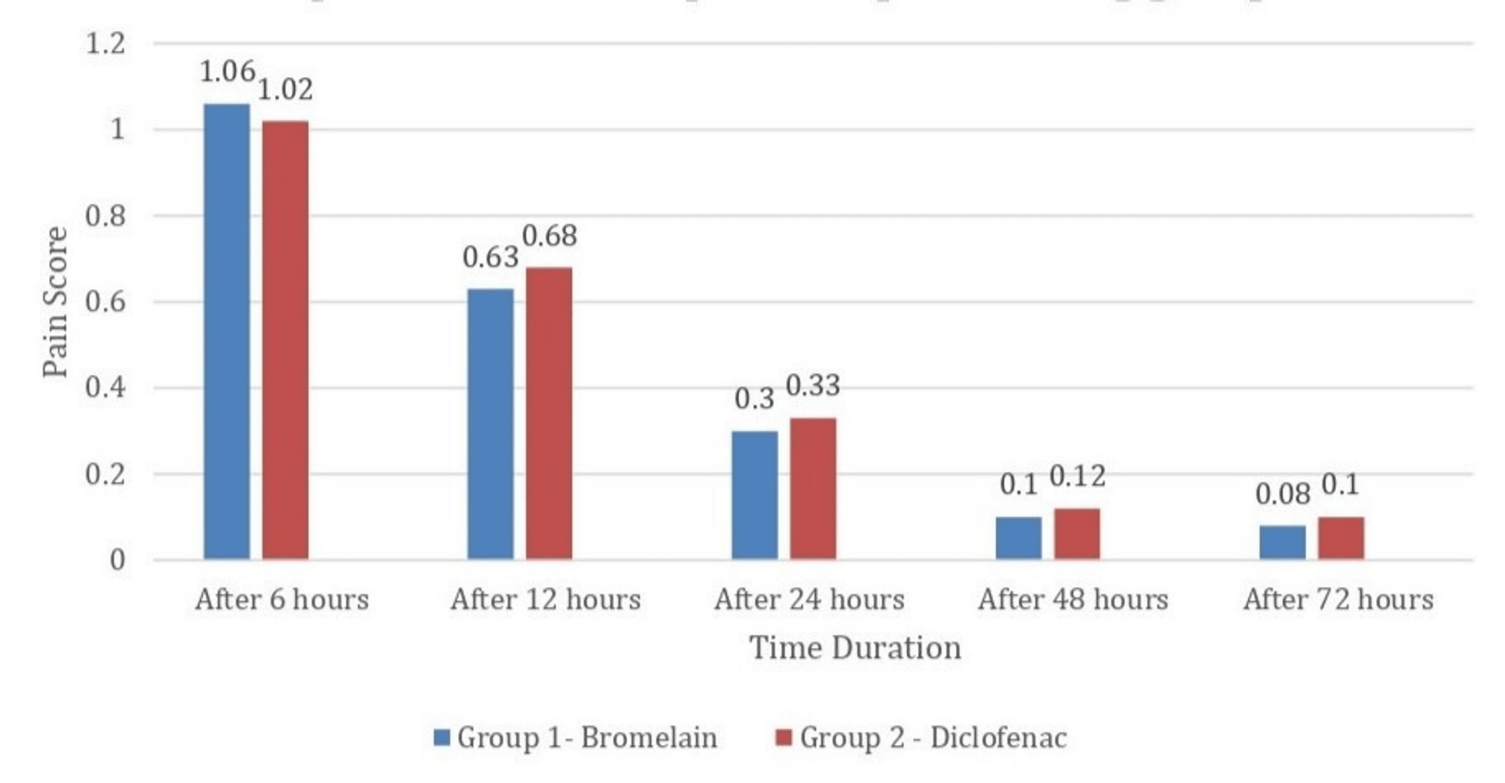
Comparison of postoperative pain among groups at different time intervals. At 6 hours (p-value=0.003*), 12 hours (p-value=0.004*), 24 hours (p-value=0.007*), 48 hours (p-value=0.78), and 72 hours (p-value=0.82). The figure was drawn by the authors of this article.

Figure 2 represents the comparison of postoperative pain scores between the two groups after 6, 12, 24, 48, and 72 hours. It was observed that patients in the diclofenac group had a lesser pain score after 6 hours in contrast to the bromelain group. However, the bromelain group showed a reduction in the intensity of pain that was comparable to the diclofenac group in 12, 24, 48, and 72 hours with no statistically significant difference.

**Figure 3:**
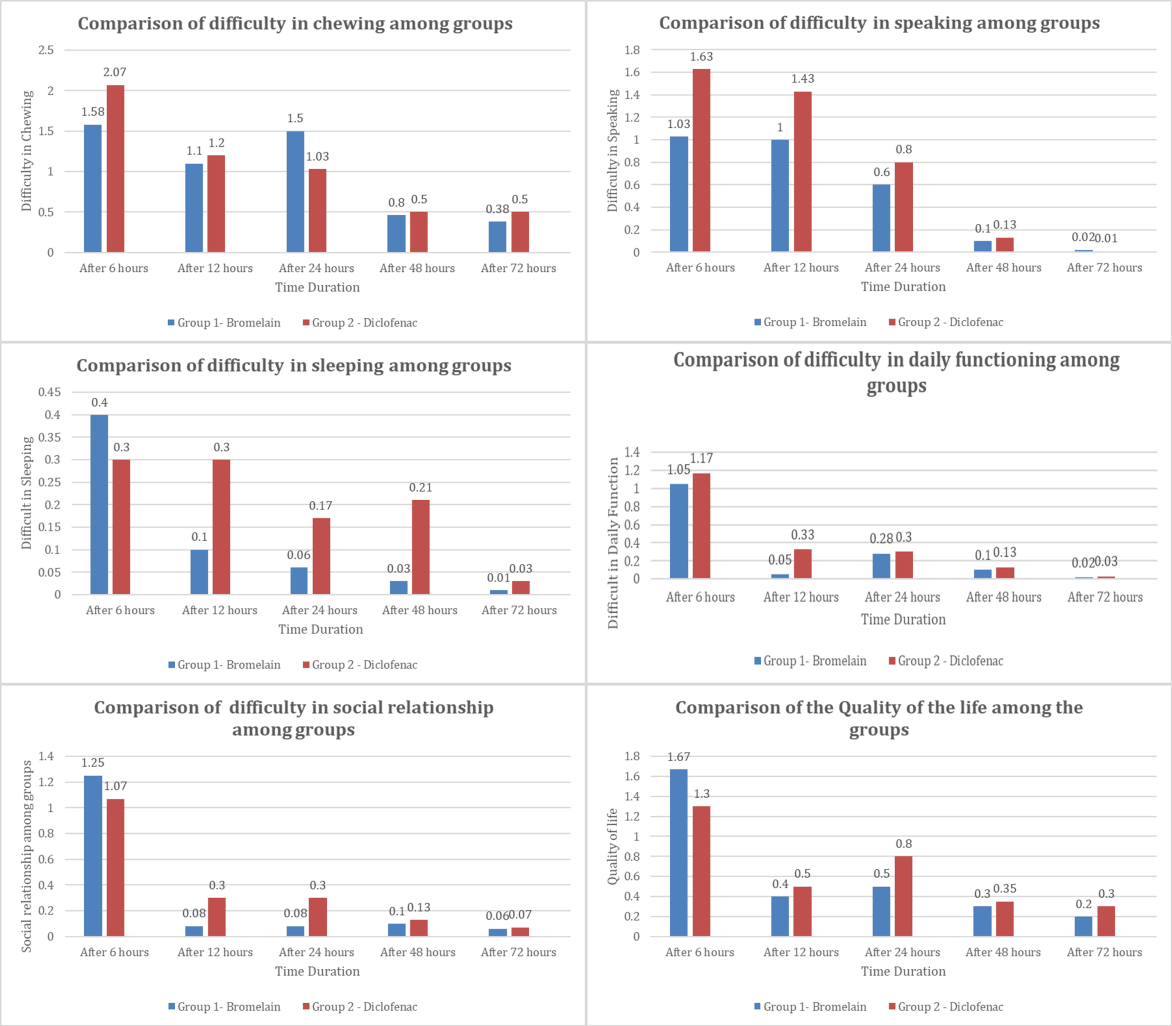
Measurement of postoperative quality of life between groups at different time intervals (Group I - bromelain and Group II- diclofenac). The overall quality of life was slightly better for the bromelain group with no statistical significance (p-value >0.5). The figure was drawn by the authors of this article.

The comparison of quality of life between the two groups based on speaking, chewing, sleeping, carrying out daily functions, maintaining social relations, and overall quality of life is illustrated in Figure 3. The bromelain group was slightly better in terms of difficulty in chewing and speaking and maintaining social relations. Nevertheless, there were no significant differences in the quality of life between both groups for any of the parameters reported.

Secondary outcomes

**Figure 4:**
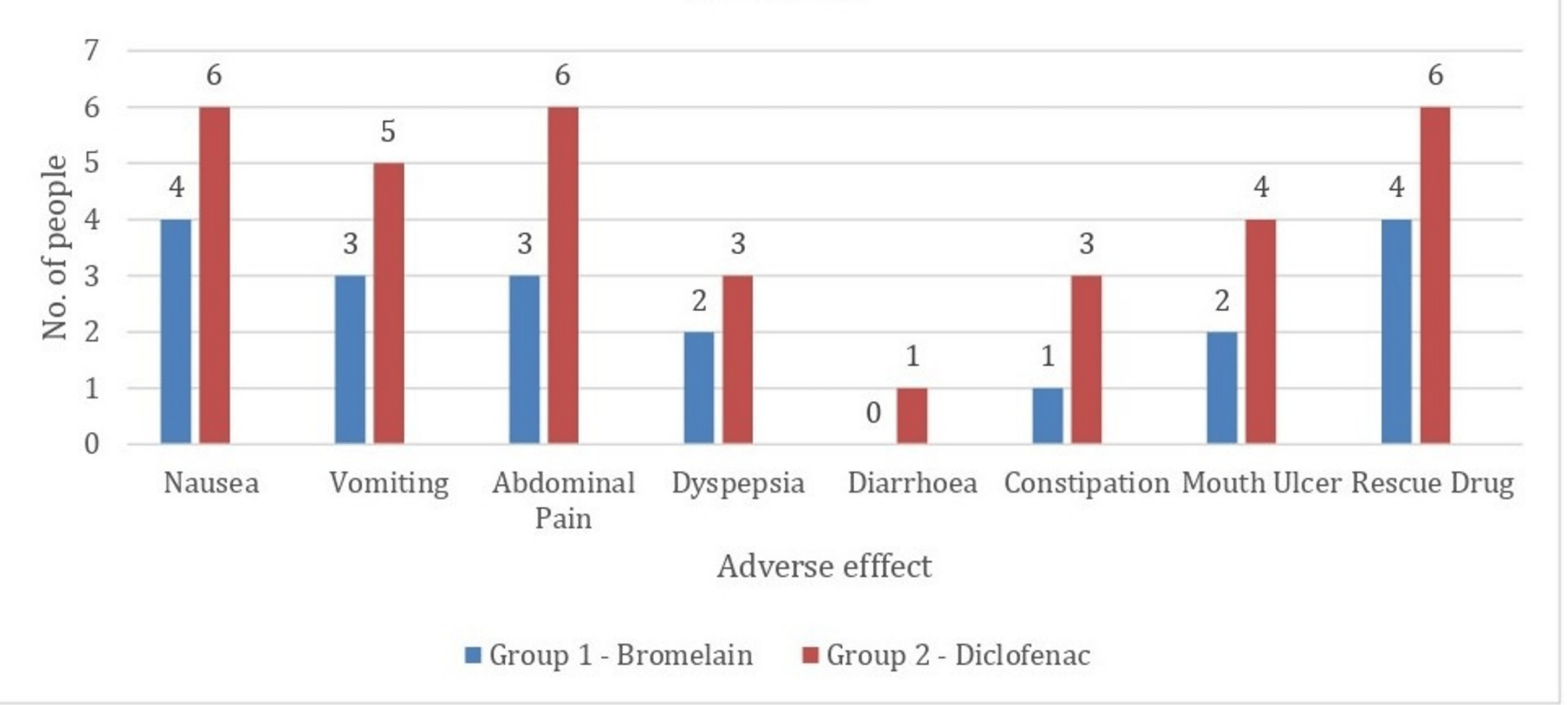
Number of patients with adverse effects and need of rescue drugs in Group I (bromelain) and Group II (diclofenac). More number of patients reported adverse effects with diclofenac as compared to bromelain at all-time intervals. The figure was drawn by the authors of this article.

The adverse effects in root canal therapy patients after 6, 12, 24, 48, and 72 hours are represented in Figure 4. There were less adverse effects recorded in the bromelain group i.e. nausea (Group I = 9%, Group II =14%), vomiting (Group I= 7%, Group II= 12%), abdominal pain (Group I=7%, Group II= 14%), dyspepsia (Group I=5%, Group II= 7%), diarrhea (Group I= 0%, Group II= 2%), constipation (Group I= 2%, Group II= 7%), mouth ulcer (Group I=4%, Group II=9%). Patients in the bromelain group also reported taking less rescue drugs (n=9%) compared to the diclofenac group (n=14%).

## Discussion

The alleviation of pain following root canal therapy remains a significant concern for patients and clinicians alike. Though a pain-free postoperative period is desired by patients, the occurrence of adverse effects considerably impairs the acceptance of any pain relief medication. In this study, the effectiveness and adverse effects of oral bromelain compared to diclofenac in alleviating pain and enhancing quality of life after root canal therapy were investigated.

Bromelain is described as an enzyme complex produced from the stems and fruits of pineapple (Ananas comosus) [13]. Bromelain has been demonstrated to have a number of advantages in experimental and clinical studies, including accelerating wound healing, lowering pain, and debriding burns [14]. Bromelain has been used in dental treatments but has not been thoroughly researched as a potential alternative therapy for pain control. It undergoes absorption in high molecular form through the intestines and is physiologically active for 6-9 hours at plasma concentrations of 2.5-4 ng/ml. Research suggests that in persons with inflammation, oral bromelain reduces plasma kinin, prostaglandin E2, bradykinin, and thromboxane B2 levels in a dose-dependent manner [6]. Moreover, it is considered non-toxic and causes fewer gastrointestinal side effects compared to commonly used NSAIDs [15].

For the assessment of postoperative pain, the method should be valid, reliable, and yield accurate and consistent results over time. VAS is the most frequently used method for the evaluation of severity of pain. It provides practical and sensitive results and is easy to analyze; hence it was used in our study [12,16]. We also assessed the impact of bromelain and diclofenac on postoperative quality of life using the POQoL questionnaire. Quality of life encompasses various physical, emotional, and social aspects of well-being, making it an important outcome measure in assessing the overall patient experience [17].

Based on our results, both medications effectively relieved postoperative pain, demonstrating their suitability for pain management in patients undergoing root canal therapy. Diclofenac provided better pain relief within the first 6 hours after therapy, whereas bromelain showed comparable pain relief efficacy after 12 hours. This finding suggests that bromelain while having comparable efficacy in relieving mild to moderate pain, has lesser efficacy during the immediate postoperative period when tackling moderate to severe pain in comparison to diclofenac (Figure 2). The overall postoperative quality of life, however, was marginally better for bromelain though there was no statistically significant difference between the groups (Figure 3).

The findings related to postoperative pain reduction align with previous research indicating the analgesic properties of both bromelain and diclofenac in various clinical settings [18,19]. In two recent studies, the effect of bromelain’s analgesia was similar to that of the diclofenac group which was used as the control [18,19]. In addition, in two other studies, bromelain exhibited an analgesic effect that was comparable to diclofenac following extractions of the third molar [11,20]. Furthermore, bromelain was shown to be effective in reducing postoperative pain in a comprehensive study and meta-analysis [21].

The bromelain group demonstrated fewer adverse effects compared to diclofenac, particularly noteworthy given the well-documented adverse effects associated with nonsteroidal anti-inflammatory drugs (NSAIDs) like gastrointestinal complications, cardiovascular risks, and renal toxicity (Figure 4) [5]. Bromelain, being a naturally derived proteolytic enzyme, may offer a safer alternative for pain management, especially in patients with preexisting medical conditions or contraindications to NSAIDs. Bromelain group patients also took lesser rescue analgesic medication than diclofenac users. This finding is similar to the findings of Majid et al., which also showed that bromelain has a few adverse effects such as nausea, diarrhea, occasional gastrointestinal issues, and allergic responses [18].

As far as we are aware, no research has been done to evaluate how well bromelain works as an interappointment or post-root canal therapy analgesic. The findings of this study have important implications for clinical practice and future research. Clinicians should consider the anticipated pain intensity and previous incidents of adverse effects reported by the patient when selecting analgesic agents for postoperative pain management. Bromelain’s favorable safety profile with lesser adverse effects suggests its potential utility as a safer alternative to traditional NSAIDs in certain clinical contexts. Future research should further investigate the comparative effectiveness and safety of bromelain and diclofenac across diverse patient populations and dental procedures. Additionally, exploring the mechanisms underlying the analgesic effects of bromelain and its potential interactions with other medications could provide valuable insights into its therapeutic utility.

Limitations

This study had a sample size of 100 patients and a follow-up period of 72 hours. A larger sample size could have improved the reliability and generalizability of our findings. A longer follow-up period to record any adverse effects and evaluate the long-term efficacy and safety profile of bromelain could be carried out in future studies.

## Conclusions

Within the limitations of this study, we can conclude that bromelain and diclofenac, except at 6 hours postoperatively, are equally effective for postoperative analgesia. Diclofenac showed better pain relief at 6 hours for moderate to severe pain. Bromelain had fewer adverse effects and showed similar effects in managing pain and postoperative quality of life when compared to diclofenac. Bromelain can be considered a safe alternative to routinely used diclofenac for pain management after root canal therapy.
